# Targeting anxiety and senescence with senolytics

**DOI:** 10.18632/aging.203985

**Published:** 2022-03-27

**Authors:** Marco Raffaele, Manlio Vinciguerra

**Affiliations:** 1 International Clinical Research Center, St. Anne's University Hospital, Brno, Czech Republic; 2 Department of Translational Stem Cell Biology, Research Institute of the Medical University of Varna, Varna, Bulgaria

**Keywords:** senolytics, anxyolitics, nociceptin receptors

Cellular senescence is a stable cell cycle arrest in response to internal and external stresses. Cellular senescence has been often defined as a double-edged sword: it can inhibit tumor development by blocking proliferation of damaged cells, while increased senescent cells burden in tissues can contribute to the development and progression of a wide range of age-related diseases, including cancer, by secreting inflammatory cytokines (SASP).

To tackle this latter age-related issue, various therapeutic strategies, including clearance of senescent cells, or suppression of harmful cytokines through the use of senolytic and senomorphic agents, respectively, are being actively investigated [1]. However, the cellular phenotype that characterized senescent cells is complex and highly heterogeneous in terms of phenotype stability, pathways involved and SASP composition, in part due to the different cell type and tissue of origin and/or senescence triggering factors. These differences in senescence phenotypes make difficult the task to identify new drugs that can target a broad range of senescent cells to produce consistent effects in various pathological scenarios. Indeed, we have recently shown that a well-studied senolytic cocktail Dasatinib + Quercetin (D+Q), which was shown to improve age-related nonalcoholic fatty liver disease (NAFLD) in mice [2], was ineffective in hampering its progression toward hepatocellular carcinoma (HCC) in a murine model of disease progression, showing on the contrary a slight protumorigenic activity [3]. The latter studies highlighted the need to find alternative solutions and identify more effective senolytics. A great help was given by the application of the high throughput automatized screening (HTS) technology, which recently led to discover new senolytic drug families, demonstrating to be a reliable tool. This approach allows testing thousands of existing molecules, available in the market as chemical libraries, to be repurposed in *in vitro* senescence models [4].

In our recent report published on Geroscience [4], we tested through HTS the LOPAC®Pfizer library compounds on a human fibroblast aphidicolin-induced senescence model, identifying the [1-(1-Methylcyclooctyl)-4-piperidinyl]-2-[(3R)-3-piperidinyl]-1H-benzimidazole (MCOPPB), a nociceptin opiod receptor (NOP) agonist and a pre-clinically tested anxiolytic, as a new potent senolytic. We found that MCOPPB was effective in killing senescence cells also when the process was induced by a chemotherapeutic drug (Doxorubicin) in HCC cells. In adult mice, MCOPPB significantly reduced locomotion – as expected with the administration of anxiolytic, and reduced also the senescence cell burden in peripheral tissues – in the white adipose tissue as well as in liver. The senescence cell clarence was accompanied by mild liver stress, characterized by a low grade of steatosis, and by an increase in adipocytes diameter together with an increased trend in body weight gain [4]. We hypothesize that these accompanying effects could be largely attributed to the decreased locomotion observed in the mice after MCOPPB administration. Beyond the reduced mobility, the behavioral tests revealed an anxiolytic effect consistent with the previous studies on this NOP agonist.

Anxiety is considered a risk factor for many age-related pathologies. In contrast, preclinical studies suggested that anxiolytics and antidepressants consumption is associated to the normalization of some hallmarks of accelerated aging (such as telomere length), and exert a neuroprotective effect [5,6]. Pharmacogenetic and pharmacologic (using D + Q) clearance of senescent cells alleviated obesity-related anxiety and NAFLD in mice [7]. Altogether, these data lead us to speculate that the anxiolytic and senolytic effects of MCOPPB might be connected [4]. It is unclear whether senescent cells can induce anxiety-like behavior via systemic SASP-dependent effects or, vice versa through the immune system / neurotransmitters functioning. Peripherally derived cytokines could inhibit neurogenesis and drive anxiety and depression. However, it has been suggested that the increase of blood plasma SASP cytokines levels, induced either by direct administration or by senescence cells transplantation, is not sufficient alone to induce anxiety-like behavior in mice [7]. It is therefore possible hypothesize that anxiety is at least in part related to local senescence occurring in specific regions of the brain, such as the hypothalamus, microglia and astrocytes, rather than a systemic effect.

A potential promising way to disentangle these central and systemic effects of old and new generation senolytics, in future studies, would rely on the utilization of nanoparticles or antibodies targeting membrane receptors in specific types of senescent cells [8]. Further efforts will be needed to determine the safety and effectiveness of the different delivery options.
